# A Simulated Dosimetric Study of Contribution to Radiotherapy Accuracy by Fractional Image Guidance Protocol of Halcyon System

**DOI:** 10.3389/fonc.2020.543147

**Published:** 2021-01-25

**Authors:** Haiyang Wang, Yuliang Huang, Qiaoqiao Hu, Chenguang Li, Hongjia Liu, Xuejuan Wang, Weibo Li, Wenjun Ma, Yichen Pu, Yixiao Du, Hao Wu, Yibao Zhang

**Affiliations:** ^1^Laboratory Key Laboratory of Carcinogenesis and Translational Research (Ministry of Education/Beijing), Department of Radiation Oncology, Peking University Cancer Hospital & Institute, Beijing, China; ^2^Laboratory Key Laboratory of Carcinogenesis and Translational Research (Ministry of Education/Beijing), Department of Nuclear Medicine, Peking University Cancer Hospital & Institute, Beijing, China; ^3^Institute of Radiation Medicine, Helmholtz Zentrum München - German Research Center for Environmental Health (GmbH), Ingolstädter Landstr, Neuherberg, Germany; ^4^State Key Laboratory of Nuclear Physics and Technology, Peking University, Beijing, China; ^5^Institute of Medical Technology, Peking University Health Science Center, Beijing, China

**Keywords:** image guided radiotherapy, deformable image registration, quality assurance, cone beam CT, Halcyon

## Abstract

**Purpose:**

Frequency of conventional kV-image guidance is sometimes sacrificed to reduce concomitant risk, leaving deviations of unguided fractions unknown. MV-imaging and treatment dose can be collectively optimized on Halcyon, where fractional MVCBCT provides complete anatomic records for course-wide dose reconstruction. By retrospective dose accumulation, this work simulated the impact of imaging frequency on patient treatment dose on the platform of Halcyon.

**Methods:**

Four hundred and sixteen MVCBCT image sets from 16 patients of various tumor sites treated with radiotherapy on Halcyon were retrospectively selected. After applying the image-guided couch shifts of the clinical records, deformable image registration was performed using Velocity software, to deform the planning CTs to the corresponding MVCBCTs, generating pseudo CTs representing the actual anatomies on the treatment day. Fractional treatment dose was reconstructed on pseudo CTs for accumulation, representing the actual patient dose (D_daily_). To simulate weekly image guidance, fractional dose was reconstructed and accumulated by incorporating 1 CBCT-guided corrections and 4 laser-guided setups of each week (D_weekly_). Limited by partially imaged volumes and different organs-at-risk of various sites, only target dose-volume parameters were evaluated across all patients.

**Results:**

GTV_D98%, CTV_D98%, PTV_D90%, PTV_D95%, PGTV_D90%, and PGTV_D95% were evaluated, where Dx% means the minimal dose received by x% volume. Pairwise comparisons were made between plan dose and D_daily_, D_daily_ and D_weekly_ respectively. PGTV_D_95%_ of accumulated D_weekly_ were significantly lower than those of accumulated D_daily_ by up to 32.90% of prescription dose, suggesting that weekly-guidance may result in unacceptable under dose to the target. The broad distribution of fractional differences between D_daily_ and D_weekly_ suggested unreliable patient positioning based on aligning surface markers to laser beams, as a popular approach broadly used on conventional Linac systems. Slight target under-dose was observed on daily reconstructed results compared with planned dose, which provided quantitative data to guide clinical decisions such as the necessity of adaptive radiotherapy.

**Conclusion:**

Fractional image guided radiotherapy on Halcyon provides more reliable treatment accuracy than using sacrificed imaging frequency, which also provides complete anatomic records for deformable dose reconstruction supporting more informed clinical decisions.

## Introduction

Image guided radiation therapy (IGRT) using various imaging modalities has been broadly applied to clinics to reduce patient setup errors and associated risk of missing target for tumors at different anatomical sites (1, 2). Although guidance is desirable for every fraction to minimize the geometric and dosimetric uncertainties (3–6), conventional kV imaging frequencies are sometimes sacrificed to balance treatment accuracy and concomitant dose (7–9). Therefore, once-a-week imaging protocol has been adopted by many centers worldwide (10) including our hospital. However, the unknown anatomic and positioning deviations of the remaining fractions may induce unacceptable target under-dose (11, 12) and potential tumor recurrence (13). Existing studies of imaging frequencies focused more on geometric impact (14–17), yet analysis based on dose accumulation is missing but clinically desirable.

Benefited from incorporable MV imaging dose (18) and faster acquisition procedure (gantry rotation speed up to 4 RPM; no extra time and operations for extending and retracting supporting arms) (19), the Halcyon system (Varian Medical Systems, Palo Alto, CA) enforced image guidance before each treatment. Fractional image guidance does not only provide more confidence on setup accuracy, but also record complete anatomic information for retrospective dose reconstruction, such as using deformable image registration (DIR) method which has been validated quantitatively by our previous study (20).

To investigate the impact of imaging frequency on the target dosimetrics, this study retrospectively selected 416 sets of megavoltage cone beam CT (MVCBCT) image guided treatment data from 16 patients of various tumor sites recruited in a phase-II clinical trial of Halcyon (Varian Medical Systems, Palo Alto, CA). Applying the clinical couch shifts as guided by MVCBCT, fraction by fraction patient dose was reconstructed using DIR on the actual anatomies represented by the MVCBCT images of the treatment day, which was accumulated as the ‘true’ reference dose. To simulate the patient dose guided by weekly imaging, only one couch shift was applied to the dose reconstruction in every five treatments, and the remaining four fractions were only guided by laser. In addition to investigating the dosimetric deviations induced by setup errors due to insufficient image guidance, the impact of inter-fractional anatomic changes on target dosimetrics were also studied in parallel.

## Methods and Material

### Patient Database

Sixteen patients enrolled in a phase-II clinical trial of Halcyon(IRB#2017QX03) were retrospectively selected (aged 30–69 years, median: 53 years), and their demographic and clinical details were given in **Table 1**, including gender, tumor site, staging, prescription dose, initial tumor diameter and weight loss during radiotherapy. Target volumes, such as Clinical Target Volume (CTV) and Gross Tumor Volume (GTV), were contoured by qualified radiation oncologists according to recommendations of ICRU Report 71 (21). Planning Target Volume (PTV) and Planning Gross Tumor Volume (PGTV) were generated by adding 3 mm (Head&Neck) or 5 mm (other sites) margins to CTV and GTV respectively. Conventional margins were used in this study to avoid potential risk of missing target, considering Halcyon-based clinical data are still limited to guide a confident reduction of margins. All targets were cross-checked by at least two experienced oncologists in accordance with our clinical protocols. Dose was prescribed to cover 95% of PTV and/or PGTV. All patients were immobilized by customized thermoplastic masks, on which the cross hairs indicating the target isocenters were marked and aligned to the onboard laser system indicating the virtual isocenter of Halcyon system. A pre-known couch shift was applied to transfer the patient from the virtual isocenter to the radiation isocenter, after which 416 MVCBCT sets were acquired in total. Automated rigid registration was conducted with necessary manual fine-tune to calculate the couch translations on the lateral, vertical and longitudinal directions respectively, as clinical corrections of setup errors, which largely considered the cough sag at the radiation isocenter induced by the shift from the virtual isocenter.

**Table 1:** Patient characteristics.

Tumor site	No	Prescription Dose(Gy/fraction)	Initial tumor diameter (cm)	Weight change (kg)
Head& Neck	1	67.87/32, 58.18/32	2.4	-14.5
	2	42.42/20, 36.36/20	10.5	-8.0
	3	50.4/28	1.9	0
	4	30/10	3.0	0
Thorax	5	54/36	9.5	0
	6	66/30, 60/30	1.9	0
	7	50/25	9.8	+1.0
	8	66/33, 60/33	5.3	-1.5
	9	66/30, 60/30	6.5	-2.0
	10	45/25	7.5	+1.0
Abdomen	11	50/25, 45/25	5.5	0
	12	50/25, 45/25	7.2	0
	13	56/28	5.8	0
Pelvis	14	45/25	13.8	0
	15	50.6/22, 41.8/22	7.0	0
	16	50.6/22, 41.8/22	6.2	-2.0

xGy/xFraction, yGy/yFraction; simultaneous integrated boosting prescriptions.

### Retrospective Dose Reconstruction

**Figure 1:**
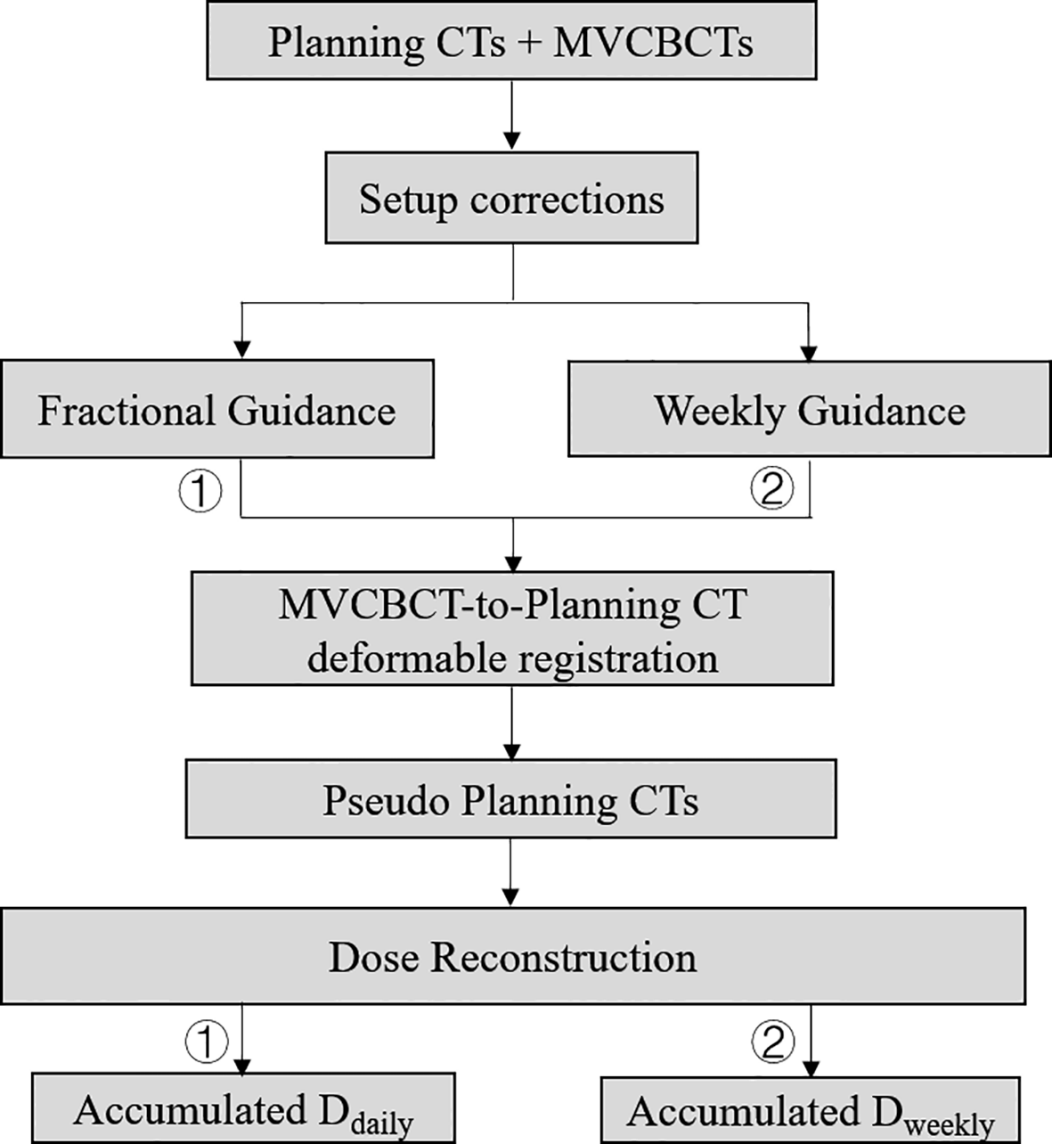
Workflow of the image registration, dose reconstruction and accumulation for comparison.

The contoured CT, treatment plans, expected dose distribution (D_plan_) and corresponding MVCBCT images were transferred from Eclipse 15.1 treatment planning system to Velocity software V4.0 (Varian Medical Systems, Palo Alto, CA) for image registration and dose reconstruction. Using both computer simulations and patient data, the accuracies of deformable image registration and dose reconstruction have been tested and validated respectively in our previous publications (22). Clinical couch shifts were used for rigid alignment between fractional MVCBCT images and the corresponding planning CTs. Using the “CBCT Corrected Multi Pass Deformable”, “CBCT Corrected Single Pass Deformable” and “Deformable Multi Pass” algorithms of Velocity, planning CTs were deformed to MVCBCTs, generating 416 pseudo CT images combining the actual anatomies on the treatment day and the CT HU for dose calculation. Planning CT images were patched with the anatomies inside the CBCT field-of-view of 27.6cm x 27.6cm x customized length. Manual cross-check was performed when necessary. Treatment dose was recalculated on these pseudo CT images, denoted as D_daily_ which represented the actual patient dose incorporating both setup corrections and anatomic changes. To simulate weekly image guidance, reverse couch shifts were applied to the last four pseudo CT images of every five fractions, simulating later-guided setup, and the dose distributions were recalculated (denoted as D_weekly_). Fractional reconstructed dose distributions were accumulated over the whole course for each patient. It took about 30 minutes to do the adaptive calculations each day per patient. The entire workflow was demonstrated in **Figure 1**.

### Dosimetric Comparison

Considering the partially imaged volume and different organs at risk of various tumor sites, only target dose was analyzed using the following parameters: GTV_D_98%_, CTV_D_98%_, PTV_D_90%_, PTV_D_95%_, PGTV_D_90%_, and PGTV_D_95%_, where D_x%_ referred to dose received by x% of the target volume. Difference of the above parameters were calculated between D_plan_ and D_daily_, representing the dose deviations induced by inter-fractional anatomic changes. Comparisons were also conducted between D_daily_ and D_weekly_, demonstrating the dosimetric impact of setup errors induced by sacrificed imaging frequency. To facilitate the comparison, all the difference values were normalized to the corresponding prescription dose, namely (D_plan_ - D_daily_)/prescription x 100%, and (D_weekly_ - D_daily_)/prescription x 100%. Analysis was performed using Wilcoxon Signed Rank Test (SPSS, 22.0) where P <0.05 was considered as statistically significant.

## Results

**Table 2** displays target statistics of plan dose, accumulated D_daily_ and D_weekly_, normalized to the corresponding prescription dose. For accumulated D_weekly_, the minimum PTV_D_95%_, PGTV_D_90%_ and PGTV_D_95%_ were only 83.77%, 81.81%, 60.77% of the prescription respectively, while the corresponding values for accumulated D_daily_ were all above 93.67% of the prescription dose. Statistically significant differences were observed between plan dose and accumulated D_daily_ in terms of CTV_D_98%_, PTV_D_90%_, PTV_D_95%_, PGTV_D_90%_, and PGTV_D_95%_ (P<0.05). Significant differences between accumulated D_daily_ and D_weekly_ were also found in PTV_D_90%_, PTV_D_95%_, and PGTV_D_95%_ (P<0.05) respectively.

**Table 2:** Target dose statistics normalized to prescription dose of 16 patients.

Parameter	D_plan_			D_daily_			D_weekly_			P1	P2
	Mean ± sth	min	max	Mean ± sth	min	max	Mean ± sth	min	max		
GTV_D_98%_	102.66 ± 1.32	100.29	105.02	98.13 ± 2.13	96.94	104.10	96.45 ± 4.44	97.73	103.83	0.245	0.177
CTV_D_98%_	103.75 ± 3.43	100.31	111.25	102.02 ± 1.94	100.07	110.88	101.36 ± 1.86	97.65	109.69	0.011	0.397
PTV_D_90%_	102.01 ± 1.55	98.59	104.08	102.88 ± 3.05	97.71	103.32	102.33 ± 3.37	93.39	103.52	0.041	0.005
PTV_D_95%_	100.14 ± 1.60	95.62	102.65	101.07 ± 1.73	94.15	100.99	100.23 ± 2.57	83.77	101.06	0.002	0.004
PGTV_D_90%_	102.04 ± 1.43	100.52	105.18	100.52 ± 2.37	96.19	103.94	98.65 ± 5.50	81.81	103.69	0.019	0.087
PGTV_D_95%_	100.98 ± 1.66	99.30	104.58	98.67 ± 2.67	93.67	103.66	94.82 ± 10.76	60.77	103.21	0.004	0.033

D_x%_, dose receiving by x% of the target volume; P1 and P2, p values of Wilcoxon Signed Rank Test over differences between accumulated D_daily_ vs. plan dose, and between accumulated D_daily_ vs. accumulated D_weekly_ respectively.

**Figure 2:**
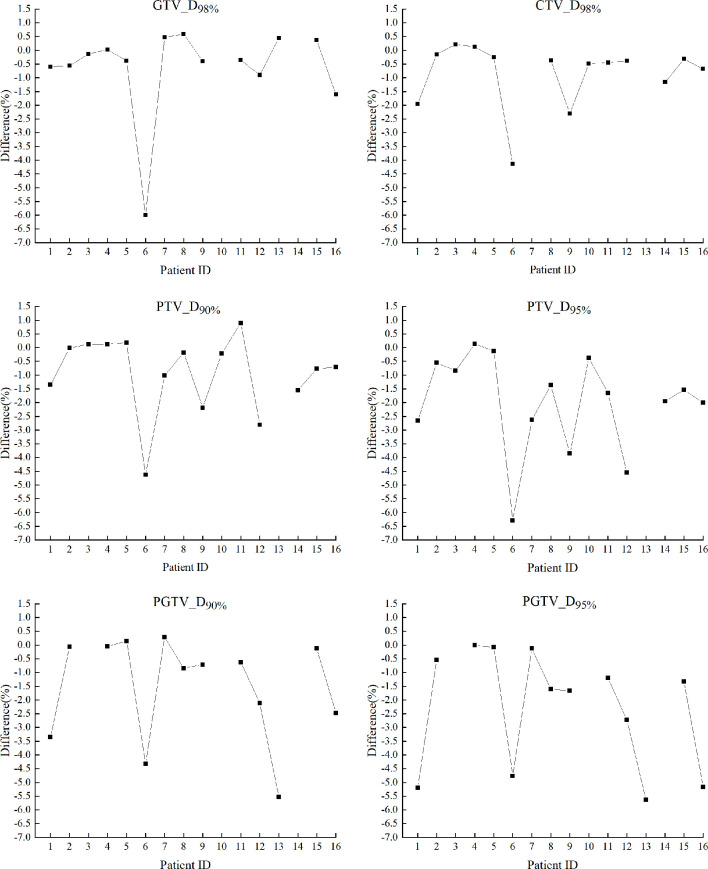
Relative differences of target dosimetrics between plan dose and accumulated D_daily_ of each patient, calculated as (D_plan_-D_daily_)/prescription x 100%. The missing structure data points were not applicable to certain patients according to the prescriptions.

**Figure 2** shows the relative differences of target dosimetrics between plan dose and accumulated D_daily_ of each patient ((D_plan_ - D_daily_)/prescription x 100%). The missing data points of some structures were due to the corresponding target volumes were not defined by physicians. In general, the accumulated D_daily_ tended to be lower than plan dose, but the magnitudes varied across patients. Site specifically, patient #1 (Head&Neck), patient #6 (Thoracic), patient #13 (Abdominal) and patient#16 (Pelvic) received relatively large under dose to the targets (>3% of prescription).

**Figure 3:**
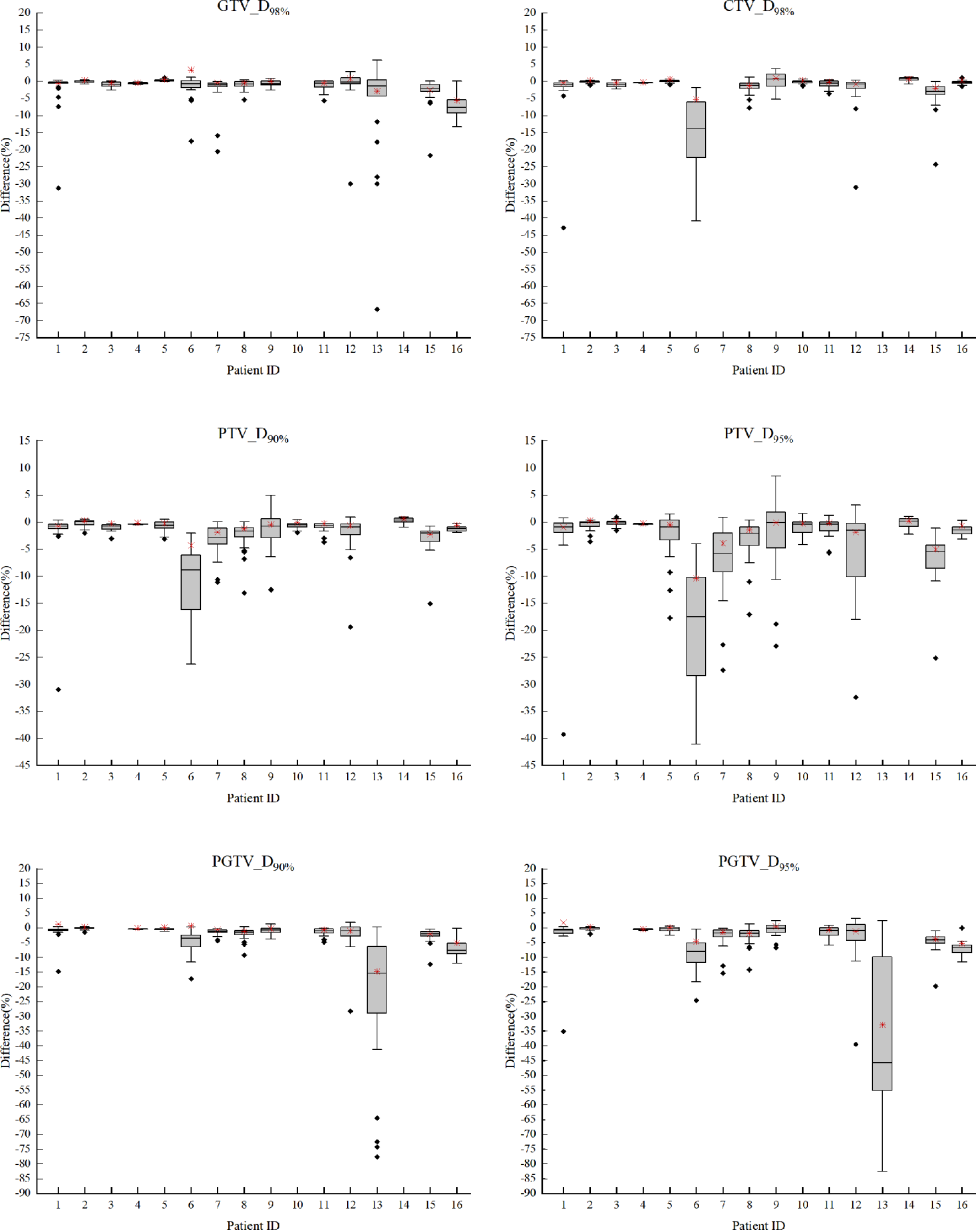
The box-and-whisker plots of relative differences of target dosimetrics between fractional D_daily_ and fractional D_weekly_ of each patient, calculated as (D_weekly_ - D_daily_)/prescription x 100%. The missing structure data points were not applicable to certain patients according to the prescriptions.

The box-and-whisker plots in **Figure 3** show the distribution of relative differences of target dosimetrics between fractional D_daily_ and fractional D_weekly_ ((D_weekly_ - D_daily_)/prescription x 100%). The accumulated differences between D_weekly_ and D_daily_ of each patient were also displayed as red crosses. For most fractions of all patients, less sufficient target dose coverage was observed in D_weekly_ than D_daily_, especially for patient #6 (Thoracic) whose median differences of fractional CTV_D_98%_, PTV_D_90%_, and PTV_D_95%_ reached -13.85%, 8.90%, and -17.52% of prescription dose respectively. The median differences of fractional PGTV_D_90%_ and PGTV_D_95%_ in patient #13 (Abdominal) reached -15.46% and -45.84%, respectively, and the corresponding accumulated differences were -14.84% and -32.90% of prescription respectively.

## Discussion

Image guidance has been widely used in radiotherapy to reduce patient setup uncertainty and monitor anatomic changes. On conventional IGRT systems, the protocol of imaging frequency varies among centers and patients to balance the accuracy, efficiency and concomitant radiological risk. Based on faster imaging and incorporable MVCBCT dose of Halcyon system, the potential benefit of dosimetric accuracy from mandatory fractional image guidance was investigated in this study.

D_daily_, the reconstructed dose based on deformable registration of fractional MVCBCT and planning CT images, accounted for both clinical couch shifts as setup corrections and deformation of patient anatomies on the treatment day, and thus reflected the actual delivered dose. Without applying four couch shifts in every five fractions (laser alignment based on surface markers only), the D_weekly_ reflected dose deviations induced by setup errors associated with simulated weekly image guidance protocol, for which fractional patient deformation was still incorporated in the dose reconstruction by using fractional MVCBCT images. Therefore, the differences of accumulated D_daily_ and D_weekly_, as shown in **Table 2**, can be considered as the net contribution of mandatory fractional imaging on Halcyon to the target dose accuracy, compared with weekly guidance protocol commonly applied on conventional IGRT systems. It should be noted that, however, potential bias may exist as a result of the differences between the imaging systems of conventional and Halcyon machines.

The differences between D_plan_ and D_daily_ as shown in **Table 2** represented the dosimetric deviations induced by patient anatomic changes on the treatment day, which also tended to cause under dose to the targets and were not correctable by image guidance alone. As a possible solution, adaptive radiotherapy (ART) could also benefit from the complete anatomic records provided by daily imaging, which is necessary for accurate dose reconstruction providing quantitative navigations for re-planning. For instance, the minimum PTV_D_95%_ and PGTV_D_95%_ of accumulated D_daily_ were only 94.15% and 93.67% of the prescription respectively, suggesting potential clinical interferences during or after the treatment course.

The comparisons between accumulated D_daily_ and D_plan_ in **Figure 2** suggested that severe target under dose induced by anatomic changes could happen for all tumor sites, including head and neck region (such as patient #1) which has been usually considered as relatively “rigid” (23). By retrospective image reviewing, noticeable anatomic changes were observed on MVCBCTs of patient #1(Head&Neck), #6 (Thoracic), #13 (Abdominal), and #16 (Pelvic) than the planning CTs, explaining their relatively larger target dose deviations. For instance, a weight loss of 14.5 kg was observed in patient #1 as shown in **Table 1**. The large inter-patient varieties of target under dose exhibited no predictable patterns, underscoring the merit of patient specific fractional dose monitoring based on DIR of three dimensional daily images, which is not possible on 2D orthogonal images although it is of less radiation dose.

As demonstrations of setup-error-induced target dose deviations as a result of different imaging frequencies, the broad distribution of fractional disparities in **Figure 3** suggested unreliable patient positioning based on aligning surface markers to laser beams, as a popular approach broadly used on conventional treatment systems. But it was also observed that fractional errors can be partially if not largely cancelled out by accumulating the dose of the whole treatment course, and weekly imaging tended to induce more severe target under dose than daily guidance. Although anatomic changes may cause considerable dose deviations in head and neck region such as patient #1 in **Figure 2** (due to severe weight loss for example), the setup-error-induced uncertainties as shown in **Figure 3** were generally smaller in head and neck patients (#1-4) than other tumor sites that were more vulnerable to deformation influences such as respiration, spine bending, or bladder filling, etc.

The possible explanations for patients exhibiting large deviations on **Figure 3** included: The large uncertainties of patient #6 (Thoracic) might be ascribable to her smallest tumor volume (initial diameter=1.9 cm) as shown in table1, suggesting a higher risk of missing small target without fractional image guidance. Large setup deviations of patient #13 (liver) might be explained by the relatively large respiratory motion at the upper abdominal region. The overall maximum accumulated dose deviation up to 32.9% of all investigated cases was also observed in this patient, if weekly imaging protocol were used. These explanatory patient features can be used to guide a personalized clinical decision making and strategy optimization in the future practice.

Although it is qualitatively expected that dosimetric accuracy can be improved by higher imaging frequencies, this study provided quantitative evidence in aspects of both anatomic changes and setup errors, based on the new Halcyon MVCBCT-guided system. It should be noticed that this work is limited by complex factors including the accuracy of dose calculation, organ deformation, setup error and MVCBCT-based DIR using B-spline mutual information algorithm. These problems are commonly observed in similar studies and are worthy of more investigations in the future. Improved image quality such as kV iterative CBCT mounted on Halcyon V2.0 may further reduce the uncertainty of DIR and dose reconstruction, which is also worthy of more studies in the future.

## Conclusion

With faster imaging acquisition and incorporable MVCBCT dose, fractional guidance protocol enforced on Halcyon system does not only improve treatment accuracy by reducing setup uncertainties, but also provide complete anatomic records for deformable dose reconstruction and quantitative guidance for informed decisions such as adaptive radiotherapy.

## Data Availability Statement

All datasets generated for this study are included in the article/supplementary material.

## Ethics Statement

The studies involving human participants were reviewed and approved by Ethnic Committee of Beijing Cancer Hospital. The patients/participants provided their written informed consent to participate in this study.

## Author Contributions

HWa, YH, and QH contributed equally to this work and were responsible for data acquisition and analyses. CL and HL contributed to performing deformable image registration. XW, WL, and WM were responsible for reviewing the data analysis result. YP contributed to the database accessibility. YD was responsible for reviewing the manuscript. HWu and YZ were responsible for designing the methodology and reviewing the whole research. All authors contributed to the article and approved the submitted version.

## Funding

This work was supported by Capital’s Funds for Health Improvement and Research (2018-2-1024, 2018-4-1027); National Natural Science Foundation of China (11505012, 11905150, 81672969, 82073333); Science Foundation of Peking University Cancer Hospital (2021-1); Fundamental Research Funds for the Central Universities/Peking University Clinical Medicine Plus X - Young Scholars Project (PKU2020LCXQ019, PKU2021LCXQ027); UICC Technical Fellowship (UICC-TF/20/722837); Peking University Health Science Center Medical Education Research Funding Project (2020YB34); National Key R&D Program of China (2019YFF01014405); Sichuan Science and Technology Program (2018HH0099). The authors declare that this study received funding from Varian Medical Systems. The funders had no role in study design, data collection and analysis, decision to publish, or preparation of the manuscript.

## Conflict of Interest

The authors declare that the research was conducted in the absence of any commercial or financial relationships that could be construed as a potential conflict of interest.
